# Implementation Strategies for Preventing Healthcare-Associated Infections across the Surgical Pathway: An Italian Multisociety Document

**DOI:** 10.3390/antibiotics12030521

**Published:** 2023-03-06

**Authors:** Massimo Sartelli, Stefano Bartoli, Felice Borghi, Stefano Busani, Andrea Carsetti, Fausto Catena, Nicola Cillara, Federico Coccolini, Andrea Cortegiani, Francesco Cortese, Elisa Fabbri, Domitilla Foghetti, Francesco Forfori, Antonino Giarratano, Francesco Maria Labricciosa, Pierluigi Marini, Claudio Mastroianni, Angelo Pan, Daniela Pasero, Marco Scatizzi, Bruno Viaggi, Maria Luisa Moro

**Affiliations:** 1 Department of Surgery, Macerata Hospital, 62100 Macerata, Italy; 2 Vascular Surgery Unit, S. Eugenio Hospital, 00100 Roma, Italy; 3 Oncologic Surgery Unit, Candiolo Cancer Institute FPO–IRCCS, 10060 Torino, Italy; 4 Anaesthesia and Intensive Care Unit, University Hospital of Modena, 41124 Modena, Italy; 5 Anesthesia and Intensive Care Unit, Azienda Ospedaliero Universitaria delle Marche, 60100 Ancona, Italy; 6 Department of Biomedical Sciences and Public Health, Università Politecnica delle Marche, 60100 Ancona, Italy; 7 General and Emergency Surgery Unit, “Bufalini” Hospital, 47521 Cesena, Italy; 8 General Surgery Unit, Santissima Trinità Hospital, 09121 Cagliari, Italy; 9 General and Emergency Surgery Unit, Trauma Center, New Santa Chiara Hospital, University of Pisa, 56100 Pisa, Italy; 10 Department of Surgical Oncological and Oral Science, University of Palermo, 90134 Palermo, Italy; 11 Department of Anesthesia Intensive Care and Emergency, University Hospital “Policlinico Paolo Giaccone”, 90134 Palermo, Italy; 12 Emergency Surgery Unit, San Filippo Neri Hospital, 00135 Roma, Italy; 13 Health and Social Services, Emilia-Romagna Region, 40127 Bologna, Italy; 14 Department of Surgery, “San Salvatore” Hospital, 61122 Pesaro, Italy; 15 Department of Surgical, Medical, Molecular Pathology and Critical Care Medicine, University of Pisa, 56126 Pisa, Italy; 16 Global Alliance for Infections in Surgery, 62100 Macerata, Italy; 17 General and Emergency Surgery Unit, S. Camillo-Forlanini Hospital, 00152 Roma, Italy; 18 Department of Public Health and Infectious Diseases, Sapienza University, 00185 Rome, Italy; 19 Unit of Infectious Diseases, ASST Cremona, 26100 Cremona, Italy; 20 Department of Medicine, Surgery and Pharmacy, University of Sassari, 07100 Sassari, Italy; 21 Department of Emergency, Anaesthesia and Intensive Care Unit, AOU Sassari, 07100 Sassari, Italy; 22 General Surgery Unit, Santa Maria Annunziata Hospital, 50012 Firenze, Italy; 23 Neuro-Intensive Care Unit, Department of Anesthesiology, Careggi University Hospital, 50139 Florence, Italy; 24 Italian Multidisciplinary Society for the Prevention of Healthcare-Associated Infections, 20159 Milano, Italy

**Keywords:** healthcare-associated infections, antimicrobial resistance, infection prevention and control, surgical infections

## Abstract

Healthcare-associated infections (HAIs) result in significant patient morbidity and can prolong the duration of the hospital stay, causing high supplementary costs in addition to those already sustained due to the patient’s underlying disease. Moreover, bacteria are becoming increasingly resistant to antibiotics, making HAI prevention even more important nowadays. The public health consequences of antimicrobial resistance should be constrained by prevention and control actions, which must be a priority for all health systems of the world at all levels of care. As many HAIs are preventable, they may be considered an important indicator of the quality of patient care and represent an important patient safety issue in healthcare. To share implementation strategies for preventing HAIs in the surgical setting and in all healthcare facilities, an Italian multi-society document was published online in November 2022. This article represents an evidence-based summary of the document.

## 1. Introduction

Healthcare-associated infections (HAIs) are infections acquired by patients while receiving healthcare. These infections are often preventable and represent a common adverse event in the healthcare system [1].

The most common HAIs include [2]: surgical site infections (SSIs), catheter-associated urinary tract infections (CAUTIs), central-line-associated bloodstream infections (CLABSIs), hospital-acquired pneumonia (HAP), ventilator-associated pneumonia (VAP), and *Clostridioides difficile* infection. 

HAIs can increase hospital mortality and morbidity, lengthen hospital stays, and increase healthcare costs. Additionally, bacteria are becoming more and more resistant to antibiotics, making the prevention of HAIs crucial to combat the spread of antimicrobial resistance (AMR). AMR is one of the greatest threats to public health worldwide and reducing the incidence of infection through infection prevention measures is one of the five actions proposed by the World Health Organization (WHO) Global Action Plan on AMR [3]. In November 2022, the European Centre for Disease Prevention and Control (ECDC) published a document about the burden of AMR in the European Union and European Economic Area (EU/EEA) from 2016 to 2020. The document estimated statistically significant increasing trends in the number of infections, attributable deaths, and disability-adjusted life years per 100.000 population caused by antibiotic-resistant bacteria [4]. 

In 2016, the ECDC estimated that, based on data from 2011 to 2012, the burden of the six main types of HAIs expressed in disability-adjusted life years in the EU/EEA was higher than the combined burden of all other 32 communicable diseases surveilled by the ECDC [5]. 

Two-point prevalence surveys on HAIs and antibiotic use were organized by the ECDC from 2016 to 2017. In 2018, the results of these surveys were published [6], reporting a total of 19,626 HAIs in 18,287 patients with HAIs in the EU/EEA. The prevalence of patients with at least one HAI sample was 5.9%. The prevalence ranged from 4.4% in primary care hospitals to 7.1% in tertiary care institutions [6]. Patients admitted to intensive care units (ICUs) had the highest prevalence, with 19.2% of patients having at least one HAI [6]. 

Many HAIs can be avoided [7]. Therefore, they should be considered an important indicator of the quality of patient care and an important patient safety issue in healthcare [8]. Surgical patients may present risk factors for the acquisition of HAIs [2]. They are more susceptible to the severe consequences of HAIs. SSIs represent the most common HAIs in surgical patients. They can result in adverse patient outcomes, including prolonged hospital stays and higher related morbidity and mortality. The risk of SSIs can be reduced by better adherence to evidence-based-prevention interventions [9].

Recently, the results of a prospective international cohort study including adult patients with hospital-acquired bloodstream infections managed in ICUs from June 2019 to February 2021 (EUROBACT-2 international cohort study) [10] were published. Hospital-acquired bloodstream infections were most frequently caused by Gram-negative bacteria, including carbapenem-resistant and difficult-to-treat bacteria. The occurrence of AMR led to delays in appropriate antibiotic treatment. The results of the study showed high mortality (37,1%). Moreover, only 16,1% of the patients were discharged alive from the hospital within 28 days. Both enhancing infection prevention and control (IPC) and implementing antimicrobial stewardship are essential to prevent HAIs and limit the spread of AMR in ICUs [11]. 

IPC is a pivotal evidence-based approach that aims to prevent the occurrence and spread of HAIs within healthcare facilities. IPC is an essential component of all healthcare systems, affecting the safety of patients. An established culture of correct healthcare practices should always control the dissemination of HAIs. The engagement of HCWs in IPC and patient safety practices can often clash with complex organizational environments, especially in settings with poor resources, and HCWs’ activity is constantly squashed by other demands. Nevertheless, as new challenges are arising and the threats of AMR are increasing, it is very important to understand how IPC strategies may be implemented.

Evidence-based guidelines can support best clinical practice. Various international guidelines for the prevention of SSIs [12,13,14], CA-UTIs [15,16,17], VAP [18], CLABSIs [19,20], and *Clostridioides* difficile infection [21] have been published in recent years.

Overcoming barriers to evidence-based-practice implementation by promoting compliance with recommended measures, and thus translating evidence into clinical practice, is crucial [22,23]. 

In 2016, the WHO published evidence-based guidelines on the core components of effective IPC programs both at the national and hospital level [24,25]. Eight core components were identified to summarize the IPC strategies. 

Regarding the prevention of SSIs, Ariyo et al. in 2019 published a systematic review of the most commonly utilized implementation strategies [26]. Implementation interventions were categorized using the “four Es” approach, describing “engage”, “educate”, “execute”, and “evaluate” as the basic components of change management.

In 2018, the WHO published an implementation manual to support the prevention of SSIs at the hospital-facility level [27], explaining how the WHO Global Guidelines for the Preventions of Surgical Site Infections can be applied according to a multimodal improvement strategy within local contexts. 

Moving forward, the working group suggested a stepwise approach based on the WHO’s “cycle of continuous improvement” that can increase awareness of preventable HAIs in HCWs. 

## 2. Methods

To share implementation strategies for preventing HAIs in the surgical setting and in all healthcare facilities, an Italian multi-society document was published in November 2022 [28]. 

An extensive review of the literature was conducted using the PubMed, MEDLINE, and Google Scholar databases, limited to the English language. The first draft of the document was initially shared by a multi-society working group composed of the Italian Multidisciplinary Society for the Prevention of Healthcare-Associated Infections (SIMPIOS), the Italian Surgical Association (ACOI), the Italian Society of Anesthesia, Analgesia, Resuscitation, and Intensive Care (SIAARTI), and the Italian Society of Infectious and Tropical Diseases (SIMIT). The National Association of Doctors of Hospital Management (ANMDO), the Italian Society of Microbiology (SIM), the Italian Clinical Microbiologists Association (AMCLI), the Italian Society of Hospital Pharmacy and Pharmaceutical Services of Health Authorities (SIFO), the National Scientific Society of Infectious Risk Nurse Specialists (ANIPIO), the Italian Society of Surgery (SIC), and the Italian Society of Operating Room Nurses (AICO), after review, also adhere to the document. Even the Italian Society of Hygiene, Preventive Medicine, and Public Health (SItI), after having revised and integrated it through the GISIO-SItI, adheres to the document. The definitive document, including current knowledge and experts’ opinion, resulted in a position and consensus statement. The document was designed to share implementation strategies in the prevention of HAIs in the surgical setting, but its application can be extended to all healthcare facilities.

This article represents a summary of the Italian document for HAI prevention across the surgical pathway. It presents a stepwise approach to infection prevention and control improvement based on the following steps: (1) preparing for an action plan, (2) planning an action plan, (3) developing an action plan, (4) creating a safe climate and favoring a cultural change, (5) assessing the impact of the action plan, and (6) ensuring long-term sustainability. 

## 3. Suggested Six-Step Approach to Infection Prevention and Control Improvement

### 3.1. Preparing for an Action Plan

As many HAIs may be preventable, each hospital should have in place, and implement, measures aimed at reducing the risk of HAIs. 

In 2018, a meta-analysis of studies evaluating the results of multifaceted interventions to reduce HAIs in acute care or long-term-care settings was published by Schreiber et al. [7]. The meta-analysis showed a potential containment of HAI rates in the range of 35–55% by implementing multifaceted interventions regardless of the country’s income level.

Hospitals should systematically identify core principles that can promptly act and drive IPC as a top priority [29]. An IPC program should be in place in each hospital. 

At the level of healthcare administrators, IPC should be considered a patient safety activity and an institutional priority [30]. 

The IPC team is the core component of an IPC program. The roles of the IPC team generally include developing IPC programs, developing and disseminating evidence-based guidelines, coordinating education and training for HCWs, supporting the surveillance of HAIs, monitoring and auditing IPC practices, and promoting the implementation of multimodal strategies. Effective IPC programs require sustained financial and political support to ensure adequate human resources and to implement programs that can have an impact at the hospital level [31].

An IPC team should be led by professionals dedicated to IPC. However, it should also include HCWs directly involved in infection prevention and control measures in their areas of clinical expertise [31]. Effective teamwork in healthcare delivery can have an immediate and positive impact on patient safety [32]. 

### 3.2. Planning an Action Plan

A robust data collection system is crucial to reduce the risk of HAIs. A baseline assessment provides information on the situation to change. It provides a critical point for evaluating changes and impact, as it establishes a basis for comparing the situation before and after an intervention, and for assessing the effectiveness of the action plan.

Moreover, a baseline assessment helps to create a sense of urgency for the changes needed to improve IPC, taking into account current needs and available local resources.

Hospitals currently without adequate and proactive systems of IPC should perform analyses of existing gaps or point-prevalence surveys to evaluate the need for an action plan based on a multimodal improvement strategy. 

Multimodal strategies should be implemented to improve IPC practices. 

A multimodal strategy consists of several elements that are implemented in an integrated way to improve changing behaviors. 

The WHO recommends a multimodal improvement strategy as a core component of an effective IPC program [33]. The term “multimodal strategy” should be understood as the use of multiple approaches that in combination can influence the behavior of HCWs, impacting patient outcomes and contributing to organizational cultural change. 

Many studies have demonstrated that IPC programs by the WHO multimodal strategy have effectively reduced the occurrence of HAIs by improving hand-hygiene practices in hospital settings [34,35,36,37,38,39].

At its core, a multimodal strategy supports the adaptation of evidence-based recommendations into practice within the local context to change HCWs’ behaviors.

### 3.3. Developing an Action Plan

The occurrence of HAIs can be reduced by adhering to guidelines [40]. Guidelines can reduce unwarranted practices, translate evidence-based practices into clinical practices, and improve healthcare quality and safety. They can be used to educate and train health professionals. Guidelines are the first step to standardizing clinical practices. However, they are not always enforceable in the local context. Adapting clinical guidelines in a local context, such as local protocols, bundles, checklists, and posters, may improve acceptance and adherence to best practices.

Active involvement of the guideline’s users can lead to significant changes in practice. Translating recommendations into a local protocol or pathway specifying responsibilities for particular actions in a hospital setting is a way to engage HCWs in guideline implementation. However, adapting guidelines in a local context could weaken the integrity of evidence-based recommendations. This process should always preserve the integrity of the evidence-based recommendations, even if differences in organizational circumstances may require significant variations in recommendations. The goal should be to define a standard of being transparent, rigorous, and replicable [41]. 

Various practical tools to support guideline implementation and best practices are available. 

The ‘bundle’ strategy has become a commonly accepted and effective method to transfer best practices into routine clinical care [42]. Care “bundles” are simple sets of evidence-based IPC measures that, when implemented collectively, can improve patient outcomes. The bundle strategy is an effective way to improve the “culture” of patient safety by promoting teamwork.

Bundles used as stand-alone interventions or as part of multimodal strategies were associated with decreased rates of CLABSIs [43,44,45,46], VAP [47,48,49], SSIs [50,51], and CA-UTIs [52,53]. Moving from guidelines [12,13,14,15,16,17,18,19,20], the working group suggested the components that should be included in prevention bundles. These components are illustrated in Table 1.

A checklist is a list of actions. Although there is no great evidence in the literature, checklists have largely been considered important tools. They can include large quantities of evidence in a concise fashion and improve best practices [54,55].

Surgical checklists are a simple strategy for addressing surgical patient safety. They can potentially prevent errors from occurring during or after surgery [56].

The use of surgical safety checklists can successfully contribute to the prevention of SSIs. Patients monitored by a checklist have a lower risk of SSIs than patients not monitored by a checklist, although this finding could be related to a better quality of care in hospitals where checklists are routinely used [57,58,59].

A clinical checklist should be practical and easy to complete, and the time required to complete the checklist should not interfere with appropriate and safe patient care. Moreover, it should be reviewed frequently to update the evidence-based practice and published guidelines.

The introduction of a checklist has been demonstrated to improve adherence to best practices and reduce the frequency of infections in the specific setting of CLABSIs [60,61].

Finally, posters can raise awareness of IPC and influence the attitudes of healthcare workers toward HAI prevention. Posters have traditionally been used in health as a resource to promote hand hygiene [62,63,64]. However, the effectiveness of posters in changing behavior is difficult to determine [65].

The principle on which posters are based is that they may act as environmental cues, engaging unconscious decision-making processes, and leading to prompt behavioral change. It is proposed that the effectiveness of posters may depend on numerous factors such as their design, content, placement, and length of placement [66]. 

### 3.4. Creating a Safe Climate and Favoring a Cultural Change

HCWs should be prepared to address complex systems and lead such systems to protect the best interests of patients. On an individual level, every HCW should have the necessary knowledge, skills, and abilities to implement effective IPC practices and should be responsible for their respective contributions to patient care. However, the engagement of HCWs in IPC and patient safety practices clashes with complex organizational environments where resources are most often inadequate and HCWs’ activity is constantly squashed by other demands. Hospitals should have regular educational programs on IPC.

Education for HCWs is the most commonly employed strategy for translating evidence-based measures into practice. Education in IPC should begin at the undergraduate level and be consolidated with adequate training throughout the postgraduate years. 

Increasing knowledge may influence the perceptions of HCWs and motivate them to change their behavior. Moreover, it is very important to persuade HCWs that IPC principles should be integrated into the concept of patient safety. Best practices are effective evidence-based procedures performed by HCWs in a given context. However, this context may be influenced by cultural, contextual, and behavioral determinants that influence clinical practice. Scientific evidence alone is not sufficient to promote behavioral change. This is because individuals within hospitals need to align new interventions and practices with their education, beliefs, perceptions, and the context in which they work. 

A cultural shift toward increased compliance with established local protocols or bundles is strongly associated with improved outcomes [67]. Although the overall quality monitoring of processes by supervisory and local quality assurance/risk management personnel can have a beneficial effect on patient outcomes, the expert panel believes that enforced mandates alone risk getting only superficial compliance and should be associated with a cultural change. Thus, it should be very important to encourage an institutional safety culture where HCWs are persuaded, rather than compelled, to be compliant with IPC measures. Hospitals with a strong culture of safety can promote education, encourage communication, and engage their HCWs in favoring a collaborative climate [68]. In these contexts, improvements in HAI prevention may be related to the overall quality of care rather than excellence in the particular area of IPC.

Finally, identifying a local opinion leader to serve as a “champion” is important because they may integrate best clinical practices and drive colleagues into changing behaviors. Frontline HCWs with satisfactory knowledge and interest in IPC may provide feedback to the prescribers and implement change within their sphere of influence, promoting on a day-to-day basis a culture in which IPC is of high priority.

### 3.5. Assessing the Impact of the Action Plan by Surveillance and Feedback

Assessing the impact of the action plan through surveillance with timely feedback allows hospitals and clinicians to assess the effectiveness of IPC strategies. Ongoing evaluation and feedback are crucial aspects of HAI prevention. 

Surveillance includes well-defined steps, including the monitoring of an event, collection, and analysis of the data associated with the event, and timely feedback to HCWs implementing strategies to decrease the incidence of the event [69], as well as improving patients’ outcomes. Surveillance with timely feedback allows hospitals and clinicians to assess the effectiveness of IPC strategies that are implemented to decrease HAI rates [70]. 

Surveillance can be passive or active. Passive surveillance is the most common form of surveillance and is based on data from patient records. It has a low sensitivity and may lead to underreporting cases because the data quality and timeliness are difficult to control. However, passive surveillance is less expensive and may be the only feasible method of surveillance in settings lacking expertise and resources where stakeholders can be engaged and involved directly in management efforts. Active surveillance has higher specificity and sensitivity than passive surveillance and should be preferred if resources are available. However, active systematic surveillance of HAIs is challenging because active surveillance is a resource- and time-consuming activity. It requires expertise and resources.

### 3.6. Ensuring Long-Term Sustainability

Healthcare practices should be regularly monitored. Feedback from the monitoring should be given to stakeholders to promote a continuous improvement of the quality of care.

The appropriateness with which IPC measures are performed depends on both HCW’ behavior and the availability of the appropriate organizational environment and infrastructure. To improve compliance with IPC programs and ensure their long-term sustainability, the frequent assessment of working practices is crucial. 

The audit is a process of comparing actual practice with a standard which should permit the reporting of noncompliance issues of concern. Providing the results of the audit to HCWs allows them to identify where improvement is needed. 

The “Audit and feedback” strategy is widely used to assess clinical practice. The “Audit and feedback” strategy can provide objective data regarding discrepancies between current clinical practices and best practices. Demonstrating this gap can act as a call for action and can motivate healthcare workers or healthcare systems to address the gap.

Sharing the audit results and providing feedback not only with audited HCWs (individual change) but also with hospital management and senior administration (organizational change) are critical steps of IPC.

For many practices, including hand-hygiene compliance, the use of the “audit and feedback” strategy has led to small but measurable improvements [71,72,73,74]. 

Quality indicators are evidence-based measures of healthcare quality that can be used to measure the quality of care and outcomes. 

In general, structural quality indicators are used to assess the setting of healthcare, such as the structural adequacy of facilities or staffing ratios. 

However, although institutional structures are certainly important for improving quality care, it is often difficult to define a clear link between structures and clinical processes.

Process indicators are used to assess whether actions are leading to high-quality care. Process indicators are built on reliable scientific evidence and should reflect common best practices, such as adequate hand hygiene, adequate insertion practices for central intravenous catheters, or the appropriate timing of antibiotic prophylaxis in surgical patients. 

Regarding the specific setting of SSIs, in 2017, the ECDC published the updated version of a technical document (HAI-Net SSI protocol, version 2.2) [75] on the surveillance of SSIs and prevention indicators in European hospitals, proposing various structure and process indicators for SSI prevention based on the strength of available evidence and the feasibility of their collection (Table 2).

Regarding the setting of HAIs in ICUs [76], the structure and process indicators selected by the ECDC HAI-Net ICU protocol are illustrated in Table 3.

## 4. Conclusions

HAIs result in significant patient morbidity and can prolong the duration of the hospital stay, causing high supplementary costs to those already sustained due to the patient’s underlying disease. Moreover, bacteria are becoming increasingly resistant to antibiotics, making HAI prevention even more important nowadays. 

The public health consequences of AMR should be constrained with prevention and control actions, which must be a priority for all health systems of the world at all levels of care.

Overcoming barriers to evidence-based practice implementation by promoting compliance with the recommended measures, and thus translating evidence into clinical practice, is crucial. Understanding how to implement evidence-based practices is fundamental to developing an effective reduction in HAIs and consequently in AMR.

## Figures and Tables

**Table 1:** Components for the prevention bundles [12,13,14,15,16,17,18,19,20].

HAI	Components of the Prevention Bundles
CLABSI	Insertion bundle: - Maintaining maximal sterile barrier precautions. - Cleaning the skin with alcohol-based chlorhexidine. - Avoiding the femoral vein for central line insertion in adult patients. - Having dedicated staff for central line insertion. - Having available insertion guidelines (including indications for central line use) and use of checklists with trained observers. - Maintenance bundle: - Evaluating central line necessity daily. - Removing unnecessary lines promptly. - Disinfecting before manipulation of the line.
CA-UTI	Insertion bundle: - Avoiding the use of urinary catheters if not necessary. - Using a correct insertion technique to minimize contamination. - Maintenance bundle: - Maintaining a closed drainage system to avoid catheter colonization. - Assessing the daily need for indwelling urinary catheters. - Avoiding routine antimicrobial prophylaxis in patients with a urinary catheter.
VAP	Maintenance bundle: - Elevating the head of the bed to between 30 and 45 degrees. - Assessing daily readiness to extubate the patient. - Performing daily oral care with chlorhexidine. - Stopping unnecessary proton pump inhibitors. - Using subglottic secretion drainage.
SSI	PAP administration bundle: - Administering appropriate PAP. - Administering PAP within 120 min before the incision according to the pharmacokinetic profiles of the antibiotic. - Redosing the antibiotic for prolonged procedures (where duration exceeds two half-lives of the antibiotic) and in patients with major blood loss (>1.5 L). - Discontinuing antibiotics after surgery. - Perioperative measures bundle: - Avoiding hair removal and, if necessary, using electric clippers. - Using alcohol-based disinfectant for surgical site preparation. - Maintaining intraoperative glycemic control with target blood glucose levels <200 mg/dL. - Maintaining perioperative normothermia with a target temperature >36 °C.

HAI: healthcare-associated infection; CLABSI: central-line-associated bloodstream infection; CAUTI: catheter-associated urinary tract infection; VAP: ventilator-associated pneumonia; SSI: surgical site infection; PAP: perioperative antibiotic prophylaxis.

**Table 2:** Structure and process indicators for SSI prevention [75].

	Structure and Process Indicators
Hospital/unit-level	- Alcohol hand rub consumption during the previous year in surgical wards. - System for root cause analysis/review of SSIs in place in the hospital collected only as aggregated by selected surgical procedure type(s).
Perioperative antibiotic prophylaxis	- Administration of PAP within 60 min before incision (except when administering vancomycin and fluoroquinolones). - Discontinuation of PAP within 24 h after initiation of surgery.
Preoperative skin preparation	- No hair removal, or if hair removal was necessary, only clipping. - Use of alcohol-based antiseptic solutions based on CHG for surgical site skin preparation.
Other prevention indicators	- Ensuring the patient’s normothermia in the perioperative period. - Using a protocol for intensive perioperative blood glucose control and blood glucose levels monitored for adult patients undergoing surgical procedures.

SSIs: surgical site infections; CHG: chlorhexidine gluconate.

**Table 3:** Structure and process indicators for HAI prevention in ICUs [76].

	Structure and Process Indicators
Hand hygiene	- Alcohol hand rub consumption during the previous year in the ICU.
ICU staffing	- Registered nurse-to-patient ratio and nursing assistant-to-patient ratio.
Antimicrobial stewardship	- Systematic review of prescribed antimicrobials within 72 h.
Prevention of VPA	- Endotracheal cuff pressure controlled and/or corrected at least twice a day. - Oral decontamination using oral antiseptics at least twice a day. - Position of the patient not supine (direct observation).
Prevention of CLABSI	- Catheter site dressing is not damp, loose, or visibly soiled (direct observation)

ICU: intensive care unit; VPA: ventilator-associated pneumonia; CLABSI: central-line-associated bloodstream infection.

## Data Availability

Data sharing is not applicable to this article as no datasets were generated or analysed during the current study.
